# Deep learning-based algorithm for the detection of idiopathic full thickness macular holes in spectral domain optical coherence tomography

**DOI:** 10.1186/s40942-024-00526-8

**Published:** 2024-01-23

**Authors:** Carolina C. S. Valentim, Anna K. Wu, Sophia Yu, Niranchana Manivannan, Qinqin Zhang, Jessica Cao, Weilin Song, Victoria Wang, Hannah Kang, Aneesha Kalur, Amogh I. Iyer, Thais Conti, Rishi P. Singh, Katherine E. Talcott

**Affiliations:** 1 Center for Ophthalmic Bioinformatics, Cole Eye Institute, Cleveland Clinic Foundation, 9500 Euclid Ave. i32, Cleveland, OH USA; 2 Case Western Reserve University School of Medicine, Cleveland, OH USA; 3 Carl Zeiss Meditec, Inc, Dublin, CA USA; 4 Cole Eye Institute, Cleveland Clinic Foundation, Cleveland, OH USA; 5 Cleveland Clinic Lerner College of Medicine, Cleveland, OH USA

**Keywords:** Artificial Intelligence, Deep learning, Macular hole, Optical coherence tomography

## Abstract

**Background:**

Automated identification of spectral domain optical coherence tomography (SD-OCT) features can improve retina clinic workflow efficiency as they are able to detect pathologic findings. The purpose of this study was to test a deep learning (DL)-based algorithm for the identification of Idiopathic Full Thickness Macular Hole (IFTMH) features and stages of severity in SD-OCT B-scans.

**Methods:**

In this cross-sectional study, subjects solely diagnosed with either IFTMH or Posterior Vitreous Detachment (PVD) were identified excluding secondary causes of macular holes, any concurrent maculopathies, or incomplete records. SD-OCT scans (512 × 128) from all subjects were acquired with CIRRUS^™^ HD-OCT (ZEISS, Dublin, CA) and reviewed for quality. In order to establish a ground truth classification, each SD-OCT B-scan was labeled by two trained graders and adjudicated by a retina specialist when applicable. Two test sets were built based on different gold-standard classification methods. The sensitivity, specificity and accuracy of the algorithm to identify IFTMH features in SD-OCT B-scans were determined. Spearman’s correlation was run to examine if the algorithm’s probability score was associated with the severity stages of IFTMH.

**Results:**

Six hundred and one SD-OCT cube scans from 601 subjects (299 with IFTMH and 302 with PVD) were used. A total of 76,928 individual SD-OCT B-scans were labeled gradable by the algorithm and yielded an accuracy of 88.5% (test set 1, 33,024 B-scans) and 91.4% (test set 2, 43,904 B-scans) in identifying SD-OCT features of IFTMHs. A Spearman’s correlation coefficient of 0.15 was achieved between the algorithm’s probability score and the stages of the 299 (47 [15.7%] stage 2, 56 [18.7%] stage 3 and 196 [65.6%] stage 4) IFTMHs cubes studied.

**Conclusions:**

The DL-based algorithm was able to accurately detect IFTMHs features on individual SD-OCT B-scans in both test sets. However, there was a low correlation between the algorithm’s probability score and IFTMH severity stages. The algorithm may serve as a clinical decision support tool that assists with the identification of IFTMHs. Further training is necessary for the algorithm to identify stages of IFTMHs.

## Background

Idiopathic full thickness macular holes (IFTMH) are a neurosensory retina defect that occur primarily as a result of abnormalities at the vitreomacular interface (VMI). They have been documented to occur in individuals from all ages and backgrounds, although prior studies have found that IFTMH most commonly affect women in their sixth to seventh decade of life [1–3]. Early symptoms associated with IFTMH include blurriness and metamorphopsia, which can progress to central vision loss if left untreated. While some IFTMH close spontaneously, vitrectomy can resolve over 90% of cases that need surgical treatment [4]. However, IFTMH may remain open even after surgery, and associated risk factors for this include older age, larger hole size (> 400 μm) and longer duration of IFTMH [5–7].

Spectral domain optical coherence tomography (SD-OCT) is the current standard of care to assess IFTMHs as it allows detailed examination of the retina layers and the VMI. A classification of macular holes based on OCT findings has been largely applied to studies that investigate IFTMH epidemiology, natural history and surgical prognosis [5, 6, 8–13]. Therefore, prompt identification of pathologic features of IFTMHs on SD-OCT images may optimize treatment and increase rates of successful hole repair and visual gains.

In recent years, the use of deep learning (DL) methods have been widely applied in ophthalmology to automate diagnosis of diseases such as age-related macular degeneration (AMD) and diabetic retinopathy (DR) [14–17]. A model capable of identifying IFTMHs features from routine clinical SD-OCTs would not only assist with the diagnostic process and decision making, but improve workflow in general and specialized clinics. Thus, the aim of this study is to assess the ability of a previously validated B-scans of Interest DL algorithm to detect pathologic features of IFTMH in SD-OCTs and classify the IFTMH in stages of severity [18–21].

## Methods

This cross-sectional study was approved by the Cleveland Clinic Institutional Review Board and informed consent was waived due to its observational nature. It adhered to the tenets of the Declaration of Helsinki and was compliant with FDA regulations and the Health Insurance Portability and Accountability Act. The study aimed to test the DL-based, Zeiss B-Scans of Interest algorithm for the detection of IFTMH features in SD-OCT B-scans, as well as investigate the correlation between its output and the severity stages of IFTMH. For this purpose, two test datasets consisting of both controls and cases were built. Eligible patients were aged ≥ 18 years old, diagnosed with posterior vitreous detachment (PVD) (controls) or IFTMH (cases), and seen at Cole Eye Institute from January 2012 to February 2021. SD-OCTs from one eye per patient were included.

### B-Scans of interest algorithm development

The B-Scans of Interest is a DL-based algorithm developed by Carl Zeiss Meditec, Inc. whose protocol has been described in detail in prior publications [18–21]. In brief, the algorithm was trained using 76,800 SD-OCT CIRRUS^™^ 4000 and 5000 (ZEISS, Dublin, CA) B-scans obtained from multiple sites worldwide. The data set included images with signal strength higher than 7, with balanced gender distribution and randomly selected eyes. Both the training and the hold-out test sets consisted of normal scans as well as retinal pathologies, including dry and wet AMD, DR, diabetic macular edema (DME), retinal vein occlusion (RVO), macular holes and epiretinal membrane (ERM). Each individual B-scan was labeled by two ophthalmologists using an online annotation tool for quality (gradable vs. ungradable based on factors such as blocked or blurred portion of the image area, being out of focus, and the presence of artifacts caused by the scanning protocol according to grader’s discretion). Gradable scans were then labeled using the same online annotation tool for the presence of eight features: subretinal fluid (SRF), intraretinal fluid (IRF), retinal pigment epithelium (RPE) atrophy, RPE elevation, disruption of inner retinal layers, disruption of vitreoretinal interface (VRI), inner segment/outer segment disruption, and “other retinal clinical findings”. In case of disagreement between graders, the annotation was adjudicated by a US-licensed retinal specialist. B-scans were considered “abnormal” if at least one of the graders had marked at least one of the above eight features. Otherwise, B-scans were considered “normal.”

**Fig. 1:**
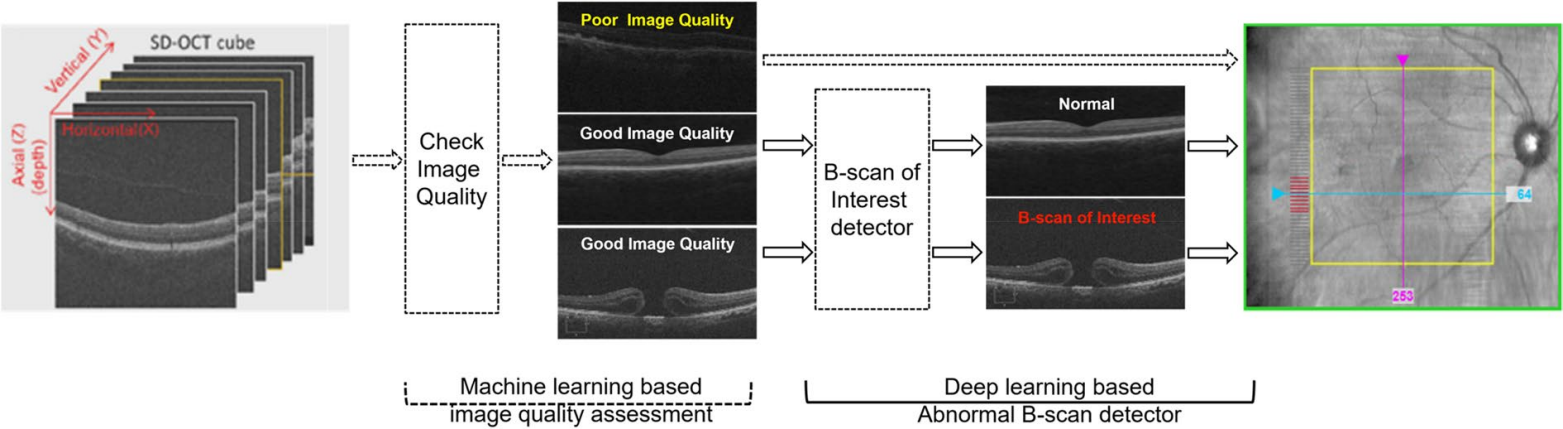
Deep learning (DL)-based algorithm for detecting image quality and identifying abnormal B-Scans of Interest. Algorithm identifies B-scans on the infrared fundus images with poor image quality (yellow) and B-scans of interest (red)

The algorithm was developed in two parts, the first assessing quality and the second detecting abnormalities in good-quality scans. The image quality assessment algorithm was first developed to identify B-scans with good or poor image quality in macular cube SD-OCT scans, and details can be found elsewhere [21]. Of these, B-scans with good image quality were used for training to automatically detect B-scans with abnormalities (i.e., any of the pathologies described above), which were called “B-scans of Interest” (Fig. 1). A 3-channel ResNet-50 neural network modified by adding inverted drop-out followed by softmax activation was the architecture used for training. The modified 3-channel ResNet-50 is pretrained on ImageNet images and was transfer trained with resized B-scans (224 × 224). The images were split into training (80%) and validation (20%) data sets at the subject level. Data augmentation was applied with rotation, horizontal flip, and vertical shift. A fivefold cross-validation model was then trained.

The algorithm provides a probability score between 0 and 1 of each individual B-scan, which classifies a B-scan as abnormal when the threshold of 0.414963 is surpassed. The primary output of the algorithm is a binary classification of normal vs abnormal B-scan (B-scans of interest). An excellent performance was achieved for the detection of B-scans of interest in both training and validation sets, with an average area under the receiving operator curve (AUC) of 0.9903 and 0.9843, respectively [21].

### Selection of images for testing the B-Scan of interest algorithm

The present study aimed to test the performance of the previously trained B-scan of Interest algorithm in detecting pathologic features in IFTMH SD-OCT B-scans using a totally different dataset. Moreover, an exploratory analysis was conducted to investigate whether there would be any correlation between the probability scores provided by the model with the stages of severity of the IFTMH. The algorithm was not retrained, nor it was originally trained to predict the severity of any diseases.

**Fig. 2:**
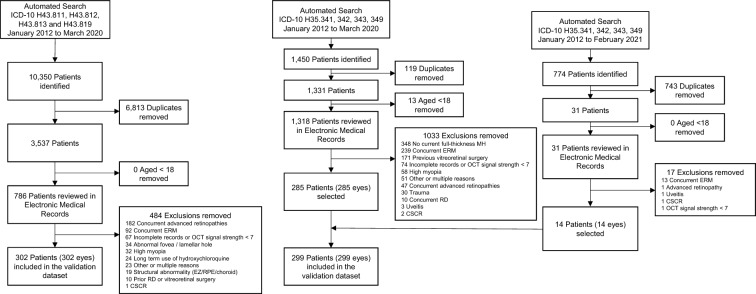
Selection of patients for control and case groups. Abbreviations: ICD-10, International Classification of Diseases 10th edition; *MH* macular hole, *ERM* Epiretinal membrane, *OCT* Optical coherence tomography, *EZ* Ellipsoid zone, *RPE* Retinal Pigment Epithelium, *RD* Retinal detachment, *CSCR* Central serous chorioretinopathy. Advanced retinopathy was defined as intermediate-stage or worse age-related macular degeneration, moderate-stage or worse diabetic retinopathy, glaucoma, retinal vein/artery occlusions and retinal dystrophies. High myopia was defined as the spherical equivalent of ≥ − 6.00 diopters or axial length of ≥ 26.5 mm. Structural abnormalities excluded were abnormal foveal contours, ellipsoid zone disruption, retinal pigment epithelium irregularity, or choroidal thickening

An automated pull of patients diagnosed with PVD (International Classification of Diseases [ICD]-10 codes H43.811, H43.812, H43.813 and H43.819) and macular holes (ICD-10 codes H35.341, H35.342, H35.343 and H35.349) between January 2012 and March 2020 was conducted to select controls and cases, respectively. The automated pull for controls and cases retrieved a total of 10,350 and 1450 patients, respectively. After removing duplicates and minors, 3537 and 1318 remained, respectively. These were randomly ordered using the Microsoft Excel random number generator function to avoid selection bias. Then, a chart review using the electronic medical records (EMR) was conducted to screen for exclusion criteria and all available SD-OCT scans were reviewed to screen for quality and confounders, as only images with signal strength over 7, and only images with an exclusive diagnosis of PVD (controls) or IFTMH (cases) were included. Secondary causes of macular holes, wrongly coded diseases and presence of concurrent pathologies that could confound results were all excluded. Whenever possible, the SD-OCT acquired at the first visit with the diagnosis was selected. It was necessary to screen 786 controls to achieve 302 eligible patients, as it was the prespecified number for this study. After screening all 1318 IFTMH patients, the sample size that matched with the size of controls was not obtained. A new automated pull with an extended period of time for searching (January 2012 to February 2021) was then conducted with the same macular hole ICD-10 codes and retrieved 774 patients, 31 of which had not been included in the first pull. The same selection process was applied. The final validation dataset included 302 controls SD-OCT macular volume cubes (38,656 B-scans) and 299 cases SD-OCT macular volume cubes (38,272 B-scans). Figure 2 details the process for selection of images.

### Annotation and image analysis for testing the B-Scan of interest algorithm

All SD-OCT macular volume cube scans (512 × 128) were acquired using the CIRRUS^™^ 4000 and 5000 (ZEISS, Dublin, CA) at the Cole Eye Institute. The scans were uploaded to the online annotation tool after being deidentified.

To build the gold-standard classification, two independent trained graders labeled each B-scan following the same process as described for the algorithm development, namely presence of SRF, IRF, RPE atrophy, RPE elevation, disruption of inner retinal layers, disruption of VRI, inner segment/outer segment disruption, and “other retinal clinical findings” [21]. Labeling discrepancies were adjudicated by a retina specialist. A different, trained grader classified each IFTMH in severity stages according to the International Vitreomacular Traction Study Group [8]. The stage classification was performed on the macular cube level using the Cirrus Review software.

The dataset was randomly split in two to test different approaches for detecting B-scans of Interest. The first one considered a B-scan to be abnormal if at least one grader annotated at least one pathologic feature (test set 1), which was the method adopted for the B-Scan of Interest algorithm development [21]. The second considered a B-scan to be abnormal if both graders agreed on the feature annotated (or after adjudication, when applicable) (test set 2), which is a more rigorous approach and less prone to human error. Microsoft Excel random number generator was used to split the data 1:1. The first half became part of test set 1, and the remaining images became part of test set 2.

### Statistical analysis

Sensitivity, specificity, accuracy and AUC were utilized as quantitative metrics to evaluate the performance of the B-scans of Interest algorithm in detection of IFTMH pathologic features. Among the features annotated, the detected ones were disruption of inner retinal layers, disruption of VRI and inner segment/outer segment disruption.

Spearman’s correlation was performed between the probability scores provided by the algorithm and severity stages of IFTMH. The probability scores are provided for each individual B-scan analyzed by the model, and therefore a compound of scores were analyzed to predict the severity stage of IFTH on the cube level.

## Results

Test sets 1 and 2 consisted of 258 cubes (33,024 B-scans) and 343 cubes (43,904 B-scans), respectively. Overall, the study cohort consisted of 405 (67.4%) females, 574 (95.5%) non-Hispanics, with a mean (SD) age of 70.5 (8.0) years. The distribution of the IFTMH severity stages was 47 (15.7%) stage 2, 56 (18.7%) stage 3 and 196 (65.6%) stage 4. Demographic characteristics are further described on Table 1.

**Table 1:** Demographic characteristics of the validation dataset

	Overall	IFTMH (n = 299)	Controls (n = 302)
Age, Mean (SD)	70.5 (8.0)	69.4 (7.1)	71.7 (8.61)
Gender, No. (%)			
Female	405 (67.4)	207 (69.2)	198 (65.6)
Male	196 (32.6)	92 (30.8)	104 (34.4)
Ethnicity, No. (%)			
Non-Hispanic	574 (95.5)	285 (95.3)	289 (95.7)
Hispanic	11 (1.8)	5 (1.7)	6 (2.0)
Missing	16 (2.7)	9 (3.0)	7 (2.3)
Eye laterality, No. (%)			
Right eye	310 (51.6)	153 (51.2)	157 (52.0)
Left eye	291 (48.4)	146 (48.8)	145 (48.0)

I*FTMH* Idiopathic full-thickness macular holes, *SD* Standard deviation

**Fig. 3:**
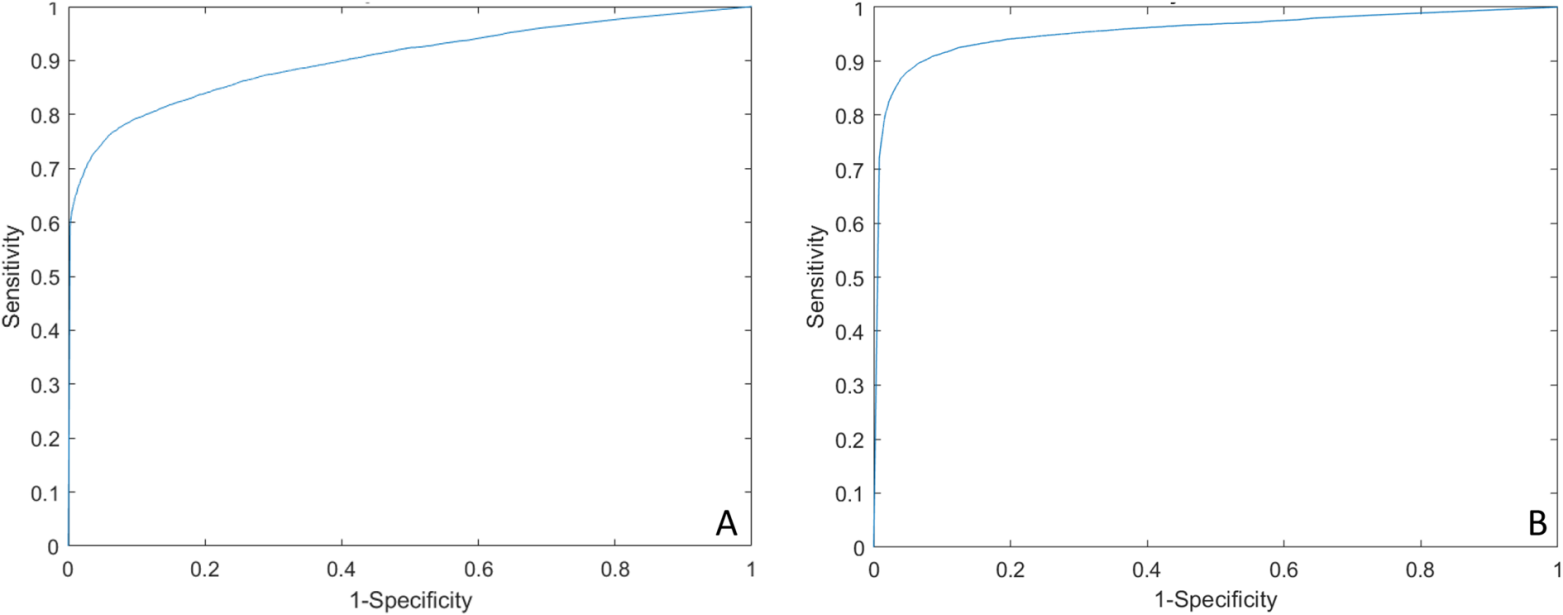
Receiving operating curves (ROC). **A** Test set 1, AUC 0.9028. **B** Test set 2, AUC 0.9555

The B-Scans of Interest algorithm achieved an average sensitivity, specificity, accuracy and AUC of 75.7%, 94.8%, 88.5% and 0.9028 for test set 1 and 89.5%, 94.2%, 91.4% and 0.9555 for test set 2, respectively (Fig. 3).

Spearman’s correlation ran on the 299 IFTMH cube scans yielded a coefficient of 0.15, suggesting a low correlation between the algorithm’s probability score and the severity stage of the IFTMH.

## Discussion

This study tested the performance of the DL-based, Zeiss B-Scans of Interest algorithm in automated identification of pathologic features of IFTMH in SD-OCT B-scans. The algorithm was tested using two datasets with different gold-standard classification methods. A high sensitivity (75.7% and 89.5% in test sets 1 and 2, respectively), specificity (94.8% and 94.2%), accuracy (88.5% and 91.4%) and AUC (90.2% and 95.5%) for detection of abnormal B-scans were achieved.

Timely diagnosis is crucial for better surgical prognosis in IFTMH cases. In busy, non-retinal specialized clinics, automated detection of possible IFTMH cases would speed triage and identify patients needing urgent care. This could improve vision outcomes, reduce medical costs and is particularly relevant in regions where ophthalmologists are not largely available. Recently, several AI-based methods of automated identification of IFTMH in fundus images and SD-OCTs have been described [22–26]. Differently from previous work, the B-Scans of Interest algorithm was developed to identify various prespecified retinal pathologic attributes in SD-OCT B-scans and characterize the individual scan as abnormal whenever any of the following is recognized: SRF, IRF, RPE atrophy, RPE elevation, disruption of inner retinal layers, disruption of VRI, or inner segment/outer segment disruption.

The B-Scans of Interest algorithm detects abnormalities on the B-scan level, which translates the algorithm’s screening applicability. In the clinical setting, if at least one B-scan of a macular cube is flagged as abnormal, there would be an opportunity for the whole cube to be reviewed by an expert, increasing the chance of subtle disease to be identified. However, this could lead to an unnecessary demand for review of macular cubes with very few abnormal B-scans with artifacts or clinically insignificant disease.

This study observed a low correlation of 0.15 between the algorithm’s probability score and the severity stage of the IFTMH on SD-OCT scans. The stages are related to size and presence of vitreomacular traction [8]. Generally speaking, the more severe the disease, or the more remarkable features it presents, the easier identification should be. In this study, one could expect that larger IFTMHs and presence of traction would translate in easier detection of disruption of retinal layers and VRI, respectively, which was not noticed. It is possible that the algorithm is so adept at picking up abnormalities that it over attributed abnormal scores to otherwise minor disruptions. Nonetheless, the B-Scans of Interest algorithm has not been specifically trained to identify severity stages of IFTMH or of any other diseases, and therefore the interpretation of the correlation results should be cautious. Further training may be required to improve this outcome.

Strengths of this study include the robust number of images used in each test dataset and the high accuracy of the algorithm achieved in test sets with different gold-standard classification methods. The IFTMH dataset was curated by trained personnel to exclude images with confounding pathologic features. Besides, the use of real-world SD-OCT images corroborates the results’ general applicability. One limitation of this study is the inclusion of exclusively idiopathic, full thickness cases of macular holes, excluding images with concurrent pathologies that would be seen in clinical practice, which limits the evaluation of the algorithm’s generalizability and applicability. The inclusion of images from just one OCT device, from one medical institution, and the cross-sectional nature of this study further limits generalizability. Another limitation is the algorithm’s primary output of normal vs abnormal B-scan only, which means the provider still needs to interpret the scans on B-scan and cube level in order to diagnose and make a decision regarding referral and treatment. Retraining the algorithm to include a more granular output (ie, pathologic feature, retinal disease, severity of disease) would be clinically valuable.

In conclusion, the DL-based, B-Scans of Interest algorithm can potentially serve as a clinical decision support tool that assists with the identification of IFTMHs. Next steps include further training of the algorithm to identify severity stages of IFTMHs. Future considerations include larger scale validation and formal review of the safety, efficacy, and reliability of the algorithm in a variety of clinical settings before implementation in clinical practice.

## Data Availability

The data that support the findings of this study are not openly available due to reasons of sensitivity. Data are located in controlled access data storage at the Cleveland Clinic.

## Abbreviations

IFTMH: Idiopathic full thickness macular hole
VMI: Vitreomacular interface
SD-OCT: Spectral domain optical coherence tomography
DL: Deep learning
AMD: Age-related macular degeneration
DR: Diabetic retinopathy
PVD: Posterior vitreous detachment
DME: Diabetic macular edema
RVO: Retinal vein occlusion
ERM: Epiretinal membrane
SRF: Subretinal fluid
IRF: Intraretinal fluid
RPE: Retinal pigment epithelium
VRI: Vitreoretinal interface
AUC: Area under the receiving operator curve
ICD: International classification of diseases
EMR: Electronic medical records
